# Supplementary material for: Genomic attributes of airway commensal bacteria and mucosa
Source: Commun Biol. 2024 Feb 12;7:171. doi: 10.1038/s42003-024-05840-3 (PMC10861553; doi:10.1038/s42003-024-05840-3)
Supplement: Supplementary file 3 — Description of Additional Supplementary Files [file 42003_2024_5840_MOESM3_ESM.pdf]

Description of Additional Supplementary Files

File name: Supplementary Data 1
Description: Bactopia report.

File name: Supplementary Data 2
Description: Genome quality report.

File name: Supplementary Data 3
Description: Kegg ontology matrix.

File name: Supplementary Data 4
Description: Kegg ontology annotations.

File name: Supplementary Data 5
Description: Community coverage by isolates.

File name: Supplementary Data 6
Description: Mucosal genomics.

File name: Supplementary Data 7
Description: Mucosal metabolomics.
